# AAV9-mediated telomerase activation does not accelerate tumorigenesis in the context of oncogenic K-Ras-induced lung cancer

**DOI:** 10.1371/journal.pgen.1007562

**Published:** 2018-08-16

**Authors:** Miguel A. Muñoz-Lorente, Paula Martínez, Águeda Tejera, Kurt Whittemore, Ana Carolina Moisés-Silva, Fàtima Bosch, Maria A. Blasco

**Affiliations:** 1 Telomeres and Telomerase Group, Molecular Oncology Program, Spanish National Cancer Centre (CNIO), Melchor Fernández Almagro 3, Madrid, Spain; 2 Centre of Animal Biotechnology and Gene Therapy, Department of Biochemistry and Molecular Biology, School of Veterinary Medicine, Universitat Autònoma de Barcelona, Bellaterra and CIBER de Diabetes y Enfermedades Metabólicas Asociadas (CIBERDEM), Madrid, Spain; Brigham and Women's Hospital, UNITED STATES

## Abstract

Short and dysfunctional telomeres are sufficient to induce a persistent DNA damage response at chromosome ends, which leads to the induction of senescence and/or apoptosis and to various age-related conditions, including a group of diseases known as “telomere syndromes”, which are provoked by extremely short telomeres owing to germline mutations in telomere genes. This opens the possibility of using telomerase activation as a potential therapeutic strategy to rescue short telomeres both in telomere syndromes and in age-related diseases, in this manner maintaining tissue homeostasis and ameliorating these diseases. In the past, we generated adeno-associated viral vectors carrying the telomerase gene (AAV9-*Tert*) and shown their therapeutic efficacy in mouse models of cardiac infarct, aplastic anemia, and pulmonary fibrosis. Although we did not observe increased cancer incidence as a consequence of Tert overexpression in any of those models, here we set to test the safety of AAV9-mediated Tert overexpression in the context of a cancer prone mouse model, owing to expression of oncogenic K-ras. As control, we also treated mice with AAV9 vectors carrying a catalytically inactive form of Tert, known to inhibit endogenous telomerase activity. We found that overexpression of Tert does not accelerate the onset or progression of lung carcinomas, even when in the setting of a p53-null background. These findings indicate that telomerase activation by using AAV9-mediated *Tert* gene therapy has no detectable cancer-prone effects in the context of oncogene-induced mouse tumors.

## Author summary

The ends of our chromosomes, or telomeres, shorten with age. When telomeres become critically short cells stop dividing and die. Shortened telomeres are associated with onset of age-associated diseases. Telomerase is a retrotranscriptase enzyme that is able to elongate telomeres by coping an associated RNA template. Telomerase is silenced after birth in the majority of cells with the exception of adult stem cells. Cancer cells aberrantly reactivate telomerase facilitating indefinite cell division. Mutations in genes encoding for proteins involved in telomere maintenance lead the so-called “telomere syndromes” that include aplastic anemia and pulmonary fibrosis, among others. We have developed a telomerase gene therapy that has proven to be effective in delaying age-associated diseases and showed therapeutic effects in mouse models for the telomere syndromes. Given the potential cancer risk associated to telomerase expression in the organism, we set to analyze the effects of telomerase gene therapy in a lung cancer mouse model. Our work demonstrates that telomerase gene therapy does not aggravate the incidence, onset and progression of lung cancer in mice. These findings expand on the safety of AAV-mediated telomerase activation as a novel therapeutic strategy for the treatment of diseases associated to short telomeres.

## Introduction

Telomeres are nucleoprotein structures localized at the ends of eukaryotic chromosomes, which are essential to protect them from degradation and end-to-end chromosome fusions. In mammals, telomeric DNA consists of TTAGGG tandem repeats bound by a 6-protein complex known as shelterin [1, 2]. Telomerase is a reverse-transcriptase able to elongate telomeres by the *de novo* addition of telomeric repeats onto chromosome ends [3]. Telomerase is composed by the telomerase reverse transcriptase catalytic subunit (TERT) and by a RNA component (*Terc*), that is used as a template for telomere elongation. Telomerase is active in pluripotent cells where it elongates telomeres within each generation, but is silenced after birth in the majority of tissues. In mice, it has been shown that both *Terc* and *Tert* are downregulated in the majority of tissues post-natally with some exceptions like the testis and the hematopoietic tissues [4–6]. In particular, adult mouse lungs, kidney, heart and brain lack detectable telomerase activity [4–6]. Thus, in the adult organism telomeres shorten associated to cellular division owing to the end-replication problem [7, 8]. When telomeres reach a critically short length this is insufficient to warrant telomere protection thus leading to the activation of a persistent DNA damage response at chromosome ends, ultimately inducing senescence or apoptosis. Several studies have shown that the presence of short/dysfunctional telomeres in the cell rather than the mean telomere length is what negatively impacts on cell division [9, 10]. Telomere shortening is considered one of the hallmarks of aging as short telomeres have been shown to be sufficient to induce organismal aging [6, 11, 12]. Even though mice are born with longer telomeres than humans they show a 100-fold faster rate of telomere shortening than humans in blood cells [13, 14]. Indeed, by using a single-cell telomere length analysis using a quantitative FISH technique, we have shown that mouse telomeres shorten with aging in all mouse tissues. In support of telomeres being rate limiting for mouse aging, first generation telomerase-deficient mice have shorter telomeres than normal and show a decreased mouse longevity, a phenotype that is anticipated with increasing mouse generations in the absence of telomerase [13, 15–21]. Thus, there is mounting evidence that although mice have on average, longer telomeres than humans, they also suffer telomere shortening with aging, and indeed this shortening is relevant for aging [13, 21].

Similar to the telomerase-deficient mouse models, human germline mutations in telomerase and other telomere-related genes are causative of the so-called telomere syndromes (ie., aplastic anemia and pulmonary fibrosis) owing to presence of much shorter telomeres than normal which lead to premature loss of the regenerative capacity of tissues [22].

Interestingly, owing to its ability to confer unlimited proliferative potential, *TERT* is also found over-expressed and mutated in the vast majority of human cancers including lung cancer where it is thought to allow cancer cell growth by ensuring a minimal telomere length to warrant telomere protection [23–31]. Telomerase is also upregulated in mouse tumors [32–34].

*Tert* transgenic mouse models with a constitutive telomerase expression in adult tissues indicated that although *Tert* over-expression does not have *per se* an oncogenic activity, its persistent expression throughout organismal lifespan could favor cancer appearance at older ages [35–38]. Of note, transgenic over-expression of *Tert* in the context of cancer-resistant mice results in longer telomeres in the adult organism and in an increased mouse longevity by 40% [39], thus demonstrating that telomerase has an anti-aging activity by virtue of its ability to maintain telomeres.

More recently, our group has also shown that reactivation of telomerase activity in adult mice by using non-integrative gene therapy vectors, which in proliferating cells, only allow temporary expression of telomerase, is sufficient to extend mouse longevity and delay many different age-related conditions, without increasing cancer incidence [6]. In particular, we used adeno-associated viral vectors (AAV) to deliver telomerase to adult tissues. These vectors present many desirable properties as they are non-integrative, show a poor immunogenicity and an excellent safety profile [40]. They are able to transduce both dividing and quiescent cells in a wide range of tissues and maintain the expression for a long time [41]. Moreover, some AAV vectors (as AAV9) have the capability of crossing the blood-brain-barrier and target brain cells upon intravenous injection in adult mice [42, 43]. In particular, expression of *Tert* using AAV9 vectors can delay physiological aging and extend longevity in wild-type mice without increasing cancer [6]. Moreover, a single treatment with AAV9-*Tert* vectors showed therapeutics effects in preventing death by heart failure after induction of myocardial infarction in mice, as well as in preventing or reversing disease in mouse models of aplastic anemia and idiopathic pulmonary fibrosis associated to short telomeres [44–46].

Importantly, AAV9-*Tert* gene therapy has not been shown to increase cancer incidence either in the context of mouse longevity studies [6] or in the context of the above-mentioned mouse models of disease owing to short telomeres [44–46]. However, mice are short lived species compared to humans, and although mice also spontaneously develop cancer with aging, a potential long-term pro-tumorigenic effect of telomerase may be missed. To circumvent this, here we set to study the safety of *AAV9-Tert* treatment in the context of cancer prone mouse models. In particular, here we tested the long-term effects of AAV9-*Tert* gene therapy in an oncogene-induced lung cancer mouse model. To this end, we tested our AAV9-*Tert* gene therapy vectors in the well-established *lox-stop-lox-K-Ras*^*G12V*^ *knock-in* mouse model in which endogenous expression of the *K-Ras*^*G12V*^ oncogene is induced upon Cre expression [47]. Our results show that AAV9-*Tert* gene therapy treatment does not affect tumor onset and/or development suggesting the safety of this therapy in the context of a cancer-prone background in mice. Interestingly, we observe that telomerase inhibition previous to the induction of the oncogene is tumor protective.

## Results

### Telomerase activation mediated by AAV9 vectors does not favor K-Ras^G12V^-induced lung carcinogenesis

**Fig 1:**
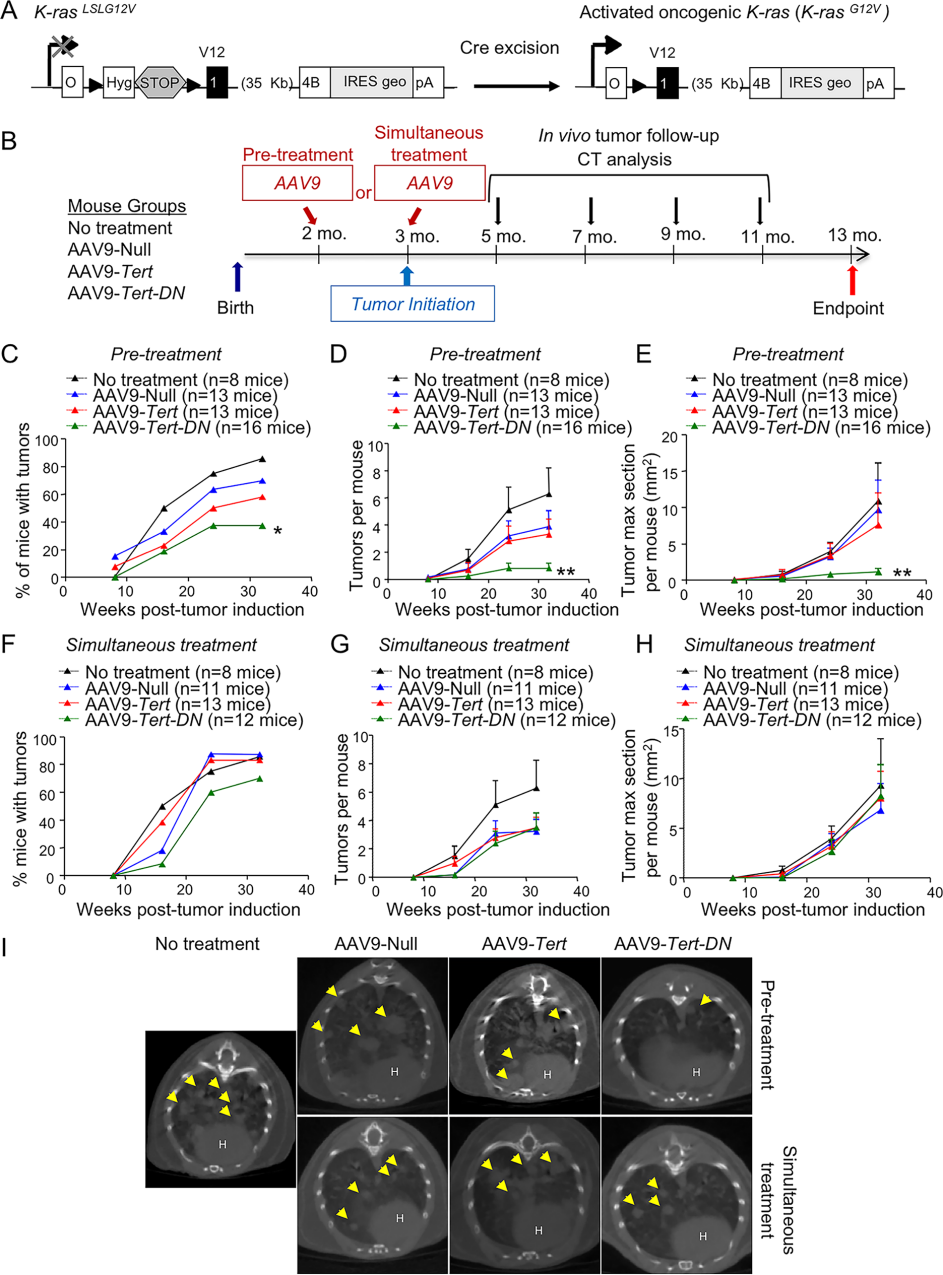
Oncogenic *K-Ras* expression and tumor follow up. **A** Genetic model. The *K-Ras*^*G12V*^ oncogene is activated after Cre-mediated excision of the STOP cassette. **B** *In vivo* approaches and imaging follow up. In the “pre-treatment” group, eight weeks old mice were transduced with AAV9 (Null, *Tert* or *Tert-DN*) vectors by tail vein injection and four weeks after they were infected with Adeno-CRE intratracheally. In the “simultaneous treatment” group, twelve weeks old mice were infected with AAV9 (Null, *Tert* or *Tert-DN*) by tail vein injection and with Adeno-CRE intratracheally at the same time. In both groups mice were analyzed every 8 weeks by computerized tomography (CT) until 32 weeks post-oncogene activation. Mice were sacrificed 40 weeks post-oncogene activation for further histological analysis. **C-H** CT quantification of the percentage of mice developing tumors (C, F), the number of tumors (D, G) and the area of the tumors (E, H) in both, pre-treated and simultaneously treated mice. **I** Representative CT images for all groups. Error bars represent standard error. *t*-test was used for statistical analysis. The number of mice are indicated in each case. Indicated p-values correspond to 32-weeks-old measurements. *, p<0.05. **, p<0,01.

Here we set out to address the impact of telomerase activation by using *Tert* gene therapy in a well-established mouse model of lung cancer initiation and progression. To this end, we used the well-established oncogenic K-Ras lung carcinogenesis model [47]. This mouse model harbors one copy of the *K-Ras*^*G12V*^ oncogene *(K-Ras*^*+/ LSLG12Vgeo*^*)* containing a STOP codon flanked by loxP sites. Expression of the Cre recombinase leads to the excision of the stop cassette and consequent expression of *K-Ras*^*G12V*^ and its *β*-galactosidase (*β-geo*) reporter (**Fig 1A**). The Cre recombinase is delivered by intratracheal instillation with replication-defective adenoviruses encoding the Cre recombinase (Adeno-Cre) [48]. The telomerase gene is packaged in adeno-associated virus type 9 (AAV9) and is delivered systemically by intravenous tail injection [6]. First, we checked whether lung cells could be co-infected with adeno-Cre and with AAV9 viruses. To this end, we simultaneously transduced mice with adeno-Cre and with AAV9 carrying GFP (AAV9-eGFP). One week after the viral transductions, mice were sacrificed and lung samples were taken for immunohistochemistry staining of *β*-galactosidase (β-geo), a surrogate marker co-expressed with the K-Ras^G12V^, and of GFP (**S1 Fig**). One week after induction of oncogenic K-Ras, *β*-Gal positive cells appear in small clusters of 4–8 cells that show a cytoplasmic foci staining [49] (**S1 Fig**). Double staining with anti-*β*-Gal (brown) and with anti-GFP (purple) of these samples revealed that these cell clusters were also positive for GFP, demonstrating that lung cells can be co-infected with adeno viruses and adeno-associated viruses (**S1 Fig**).

Next, to address the impact of AAV9-*Tert* treatment in the context of oncogenic K-Ras expression, we tested two possible scenarios. First, we used a “pre-treatment” strategy in which young 8-week-old mice were first infected by tail vein injection with either AAV9-Null, AAV9-*Tert* or a catalytically inactive AAV9-*Tert-DN* vectors. TERT-DN acts as a dominant negative and has been previously described by us to inhibit endogenous telomerase activity and to impair the growth of cancer cell lines [50, 51]. Four weeks after treatment with the viral vectors, we induced the expression of the oncogenic K-Ras by intratracheal instillation with replication-defective adenoviruses encoding the Cre recombinase (Adeno-Cre)(**Fig 1B**). In a second experimental setting, we used a “simultaneous treatment” strategy in which 12-week-old mice were treated with either one of the three AAV9 vectors (AAV9-Null, AAV9-*Tert* and AAV9-*Tert-DN*) by tail injection at the same time that they were intratracheally treated with Adeno-Cre to activate K-Ras (**Fig 1B**). In both strategies, we included a group of 12-weeks old mice that were not infected with any of the AAV9 vectors but were treated with adeno-Cre, as positive control for oncogenic K-Ras tumorigenesis in the absence of AAV9 viral vectors. Two months after oncogene activation by adeno-Cre inoculation, tumor growth was longitudinally followed by using computed tomography (CT) every two months (**Fig 1B**). Mice were sacrificed 40 weeks after oncogene activation and samples were taken for histological analysis (**Fig 1B**).

*In vivo* tumor follow-up by CT showed that AAV9-*Tert* treated mice showed the same number of mice affected with tumors as well as the same number of tumors per mouse and the same tumor area as the AAV9-Null treated mice and the untreated control group both in the “pre-treatment” and “simultaneous treatment” experimental settings (**Fig 1C–1I).** Interestingly, mice pre-treated with AAV9-*Tert-DN* vectors before oncogene activation (“pre-treatment group”), showed a significant decrease in the percentage of mice developing tumors at 32 weeks post-oncogene activation (**Fig 1C**). In addition, pre-treated AAV9-*Tert-DN* mice showed less number of tumors per mouse and a reduced tumor area compared to either AAV9-*Tert* treated, AAV9-Null treated mice or to the untreated control group at 32 weeks post-oncogene activation (**Fig 1D, 1E and 1I**). In contrast, in the “simultaneous treatment” group, we observed no significant differences between the AAV9-*Tert-DN* and the other groups both in the percentage of mice with tumors, in the number of tumors per mice or in the tumor area (**Fig 1F and 1I**).

We also studied the impact of AAV9-*Tert* gene therapy in K-Ras-induced lung tumorigenesis in a p53-deficient background, a more aggressive scenario in which lung tumors develop even more rapidly [48]. To this end, we first treated the mice with the AAV9 viruses and then activated oncogenic K-Ras (“pre-treatment” strategy; **Fig 1A**). Mice were sacrificed 5 months post-adeno-cre infection for macroscopic quantification of tumor burden (**S2 Fig**). In this p53-deficient genetic background, 100% of the experimental mouse groups developed lung tumors and no significant differences in the number of tumors and in tumor size were detected between AAV9-*Tert* treated group as compared to AAV9-null and untreated control groups (**S2 Fig**).

All together, these results clearly indicate that telomerase gene therapy has no effect in tumor onset or in tumor development in a context of oncogenic K-Ras lung tumorigenesis even in a p53-deficient background, in mice. In contrast, telomerase inhibition by using a dominant negative *Tert* gene, administered previously but not simultaneously to oncogene activation significantly impairs tumor growth.

### Telomerase activation mediated by AAV9 vectors does not increase malignancy in *K-Ras*^*G12V*^-mediated lung cancer

**Fig 2:**
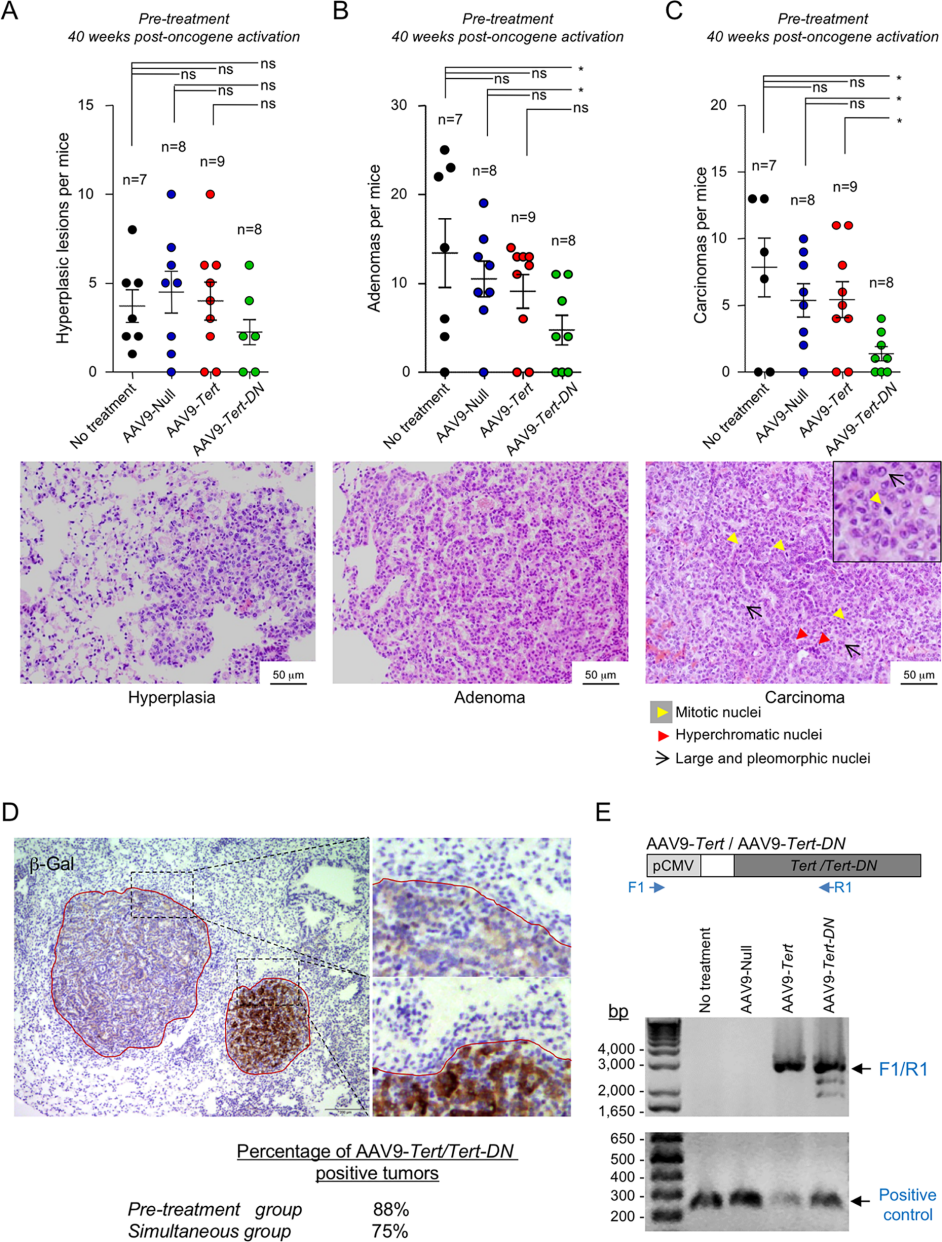
AAV9-*Tert* therapy does not aggravate *K-Ras*-mediated lung tumor progression. **A-C** Quantification of total number of hyperplasias (A), adenomas (B) and carcinomas (C) in pre-treated 40 weeks old mice. A representative image of each lesions is shown in the panels below. Hyperplasic lesions show alveolar-like structures with uniform nuclei and similar to healthy lung tissue. Adenomas contain cells with slightly enlarged nuclei with prominent nucleoli and disturb the adjacent tissue. Carcinomas present cells with very large, pleomorphic nuclei (black arrows), high mitotic index (yellow arrow head) and hyperchromatism (red arrow head). Error bars represent standard error. *t*-test was used for statistical analysis. The number of mice are indicated in each case. *, p<0.05. **D** Detection of *β*-galactosidase expression by immunohistochemistry in the lungs as a surrogate marker of oncogenic *K-Ras*^*G12V*^ expression. Note that *β*-Gal positive areas coincide with tumors (within the red line). **E** PCR detection of AAV9-*Tert* and AAV9-*Tert-DN* viral genome. The PCR reaction was performed with total tumor DNA as template and primers annealing at the 5’-end within the CMV promoter (F1) at the 3’-end within the *Tert*/*Tert-DN* ORF (R1) that renders a 2.905 kb DNA fragment. A representative agarose gel image of the PCR product from the untreated, AAV9-Null, AAV9-*Tert* and AAV9-*Tert-DN* treated tumors is shown. A PCR with primers annealing to a mouse genomic 0.3 kb DNA fragment was run as a PCR positive control. The DNA ladder is shown to the left.

To analyze the degree of malignancy of the *K-Ras*^*G12V*^ lung tumors that appeared in the different experimental cohorts, we performed hematoxylin and eosin staining in serial sections of paraffin embedded lungs at 40 weeks post-oncogene activation in the “pre-treatment” group, which was the one that showed significant differences in tumor growth in the AAV9-*Tert-DN* cohort. Lesions were classified either as hyperplasias, adenomas, or carcinomas. Hyperplasic lesions showed alveolar-like structures with uniform nuclei and similar to healthy lung tissue. Adenomas contained cells with slightly enlarged nuclei with prominent nucleoli and disturbed the adjacent tissue. Carcinomas presented cells with very large, pleomorphic nuclei, high mitotic index with aberrant mitosis, and hyperchromatism (**Fig 2A–2C**). We observed no significant differences in the total number of hyperplasic lesions between the different mouse cohorts (**Fig 2A**). Similarly, mice pre-treated with AAV9-*Tert* did not show any significant differences in the incidence of adenomas and carcinomas compared to mice treated with the AAV9-Null or to mice not treated with viruses (mock) (**Fig 2B–2C**). Interestingly, we observed a significant reduction in the number of adenomas in the AAV9-*Tert-DN* pre-treated mice compared to mock and AAV9-Null pre-treated mice (**Fig 2B**). Furthermore, the total number of carcinomas was also lower in mice pre-treated with AAV9-*Tert-DN* compared to mock, AAV9-Null and AAV9-*Tert* treated mice (**Fig 2C**). These results demonstrate that AAV9-*Tert* gene therapy does not increase the malignancy of K-ras induced lung tumors. Again, telomerase inhibition by using AAV9-*Tert-DN* gene therapy vector previous to oncogene-induction had a significant impact in decreasing both tumor onset and tumor malignancy.

To confirm that the tumors originated from cells simultaneously infected with adeno-cre and the AAV9 vectors, we first determined *K-Ras*^*G12V*^ expression in tumors by detecting the expression of its surrogate *β*-galactosidase marker by immunohistochemistry (Materials and Methods). We found that all tumors originated were from adeno-cre infected cells (**Fig 2D**). We next determined the presence of the AAV9-*Tert*/AAV9-*Tert-DN* viral genomes (vg) in the tumors at the end-point from the different experimental groups. We performed a PCR from total tumor DNA as template and using primers annealing at the 5’-end within the CMV promoter that drives the expression of the *Tert* transgene and at the 3’-end within the *Tert*/*Tert-DN* ORF (**Fig 2E**). We analyze a total of 26 tumors belonging to AAV9-Null, AAV9-*Tert* and AAV9-*Tert-DN* treated mice from the “pre-treatment” from the “simultaneous” group. We found that the AAV9-*Tert*/*Tert-DN* vg was detected in 88% of all the tumors analyzed from the “pre-treatment” and in 75% of the tumors analyzed from the “simultaneous” group (**Fig 2E**). These results clearly show that K-Ras^G12V^ induced tumors aroused from cells also infected with the AAV9 vectors (**Fig 2D and 2E**).

### AAV9-*Tert* treatment results in Tert mRNA over-expression in the lung

**Fig 3:**
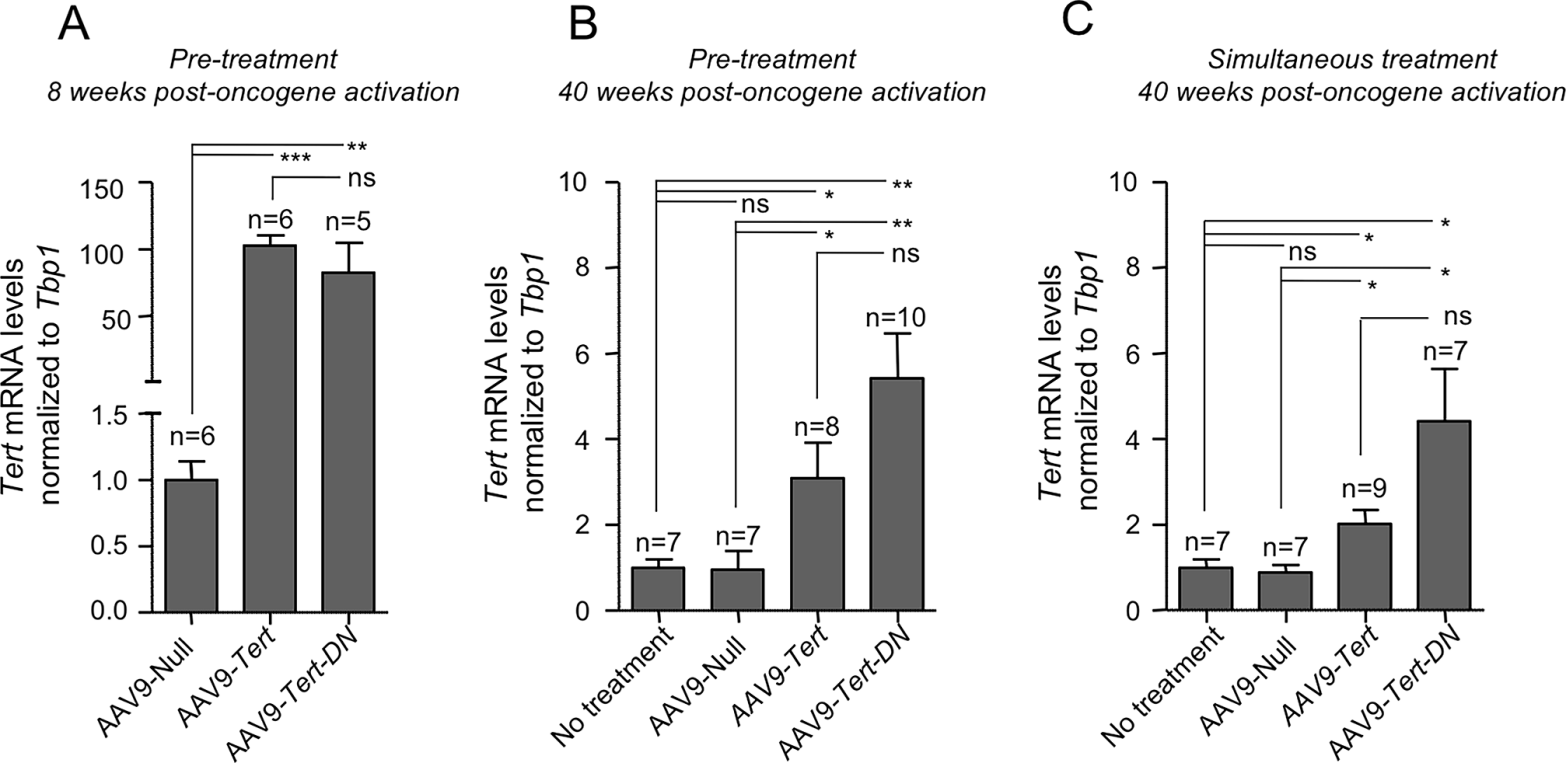
***Tert* and *Tert-DN* are over-expressed after AAV9 injection: A-C** *Tert* expression levels measured by Q-PCR in healthy lungs of pre-treated mice at 8 weeks post-oncogene activation (A) and pre-treated (B) and simultaneously treated (C) mice 40 weeks post-oncogene activation. Error bars represent standard error. *t*-test was used for statistical analysis. The number of mice are indicated in each case. *, p<0.05. **, p<0,01.

Next, to study whether the effects of the different viral vectors carrying the wild-type and mutant *Tert* genes could be related to their expression levels, we studied the transcriptional expression levels of both *Tert* and *Tert-DN* mRNAs in treated lungs both at 8 weeks and 40 weeks post-oncogene activation by using quantitative PCR (qPCR) analysis. We found a similar upregulation of both *Tert* and *Tert-DN* mRNA levels at 8 weeks after oncogene activation and this up-regulation was maintained, although to lower levels, at 40 weeks after oncogene activation in both the “pre-treatment” and “simultaneously treatment” cohorts (**Fig 3A–3C**).

### AAV9-*Tert* treatment results in longer telomeres in lung cells

**Fig 4:**
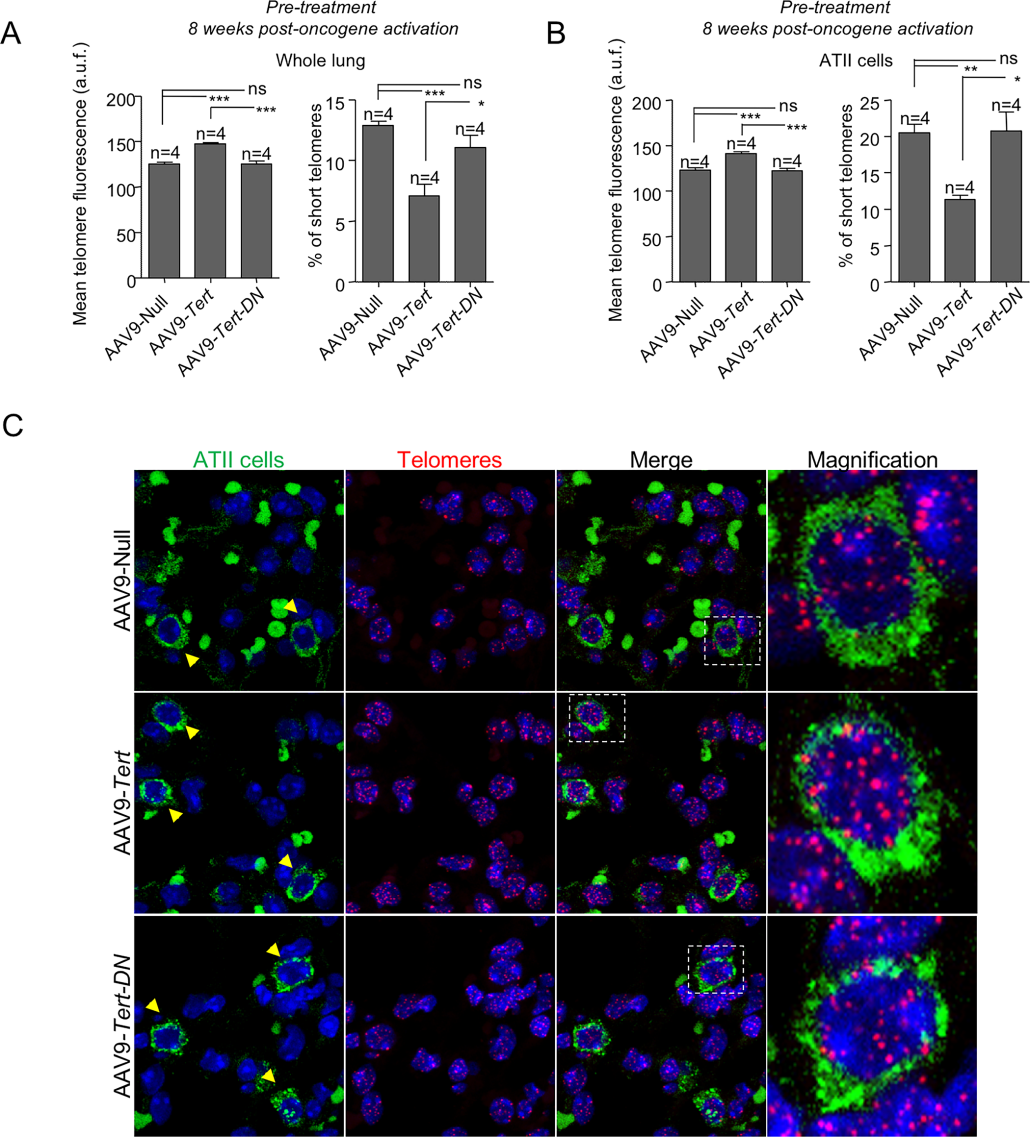
Telomerase gene therapy results in longer telomeres in lung cells. **A-B** Mean telomere fluorescence and percentage of short telomeres in whole lung (A) and in ATII cells (B) at 8 weeks post-oncogene activation in the pre-treated mice. **C** Representative images showing ATII cells stained with anti-SFTPC (green cytoplasm, yellow arrow heads) by IF and telomeres stained in red by FISH. Auto-fluorescent red blood cells lacking nuclei are observed in the images. Magnification images are shown to the right. Error bars represent standard error. *t*-test was used for statistical analysis. The number of mice are indicated in each case. *, p<0.05. **, p<0,01. ***, p<0,001.

We showed previously that AAV9 vectors target preferentially alveolar type II cells (ATII) in mouse lungs (80% of AAV9-infected lung cells are ATII cells) [46]. To address the effects of treatment with AAV9-*Tert* and AAV9-*Tert-DN* viral vectors in telomere length in whole lung tissue and specifically in ATII cells, we performed an immuno-telomereFISH using an anti-Sftpc antibody as a marker for ATII cells and a telomeric quantitative FISH probe to measure telomere fluorescence on lung sections [46]. Telomere fluorescence was measured in healthy whole-lung tissue and in ATII cells at 8 weeks post-oncogene activation in the “pre-treatment” group, which was the one that showed differences in tumor growth between the different viral treatments (**Fig 4**). The results show that AAV9-*Tert* treated lungs show significantly higher telomere fluorescence and a lower percentage of short telomeres (telomeres below 25^th^ percentile of telomere fluorescence) in both healthy whole-lung tissue and in ATII cells compared to AAV9-Null and AAV9-*Tert-DN* treated lungs (**Fig 4A–4C**).

**Fig 5:**
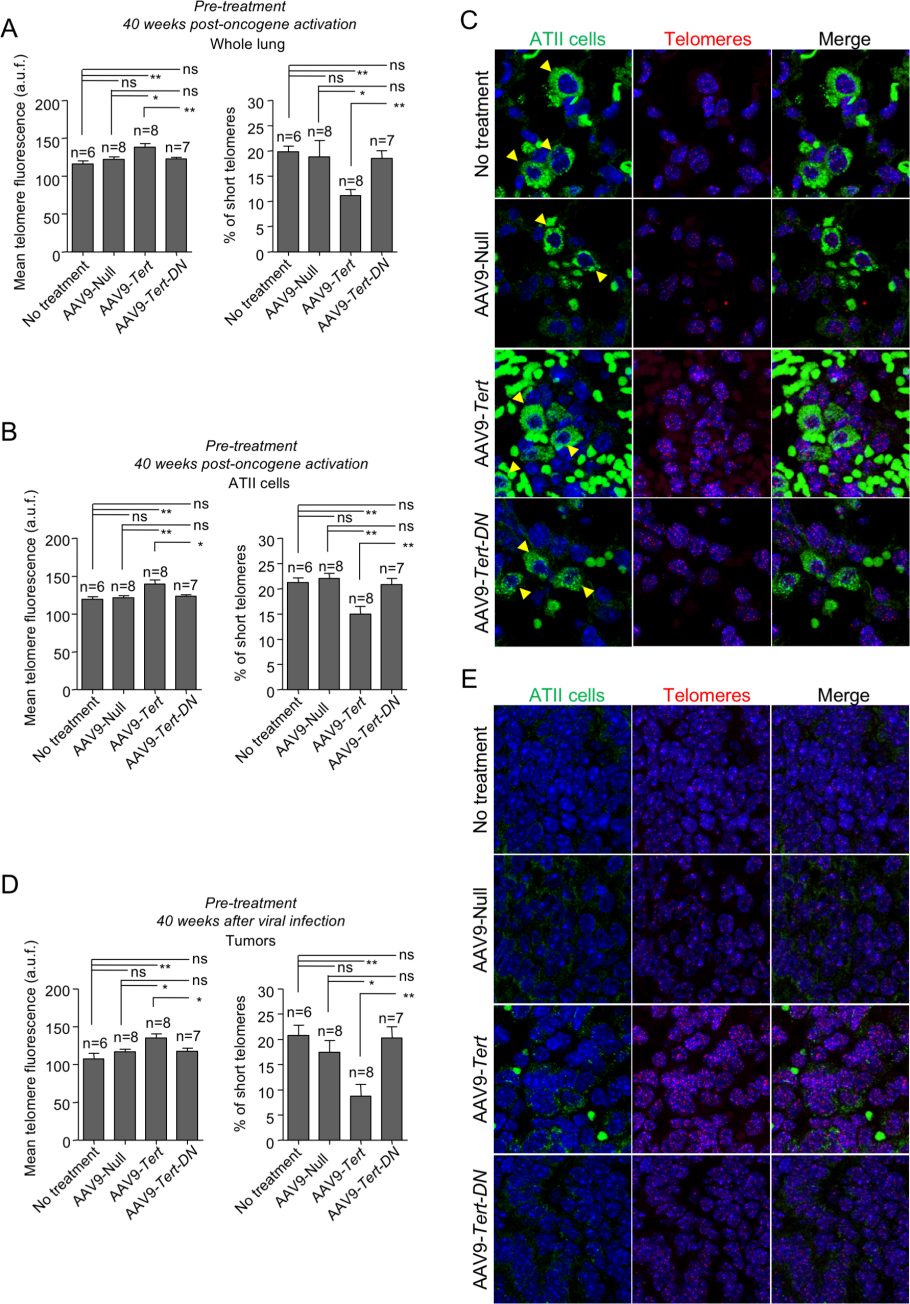
Telomerase gene therapy results in longer telomeres in lung cells and tumors in the “pre-treatment” group. **A-E** Mean telomere fluorescence and percentage of short telomeres in whole lung (A), in ATII cells (B) and in tumors (D) at 40 weeks post-oncogene activation in the pre-treated mice. **C,E** Representative images showing ATII cells stained with anti-SFTPC (green cytoplasm, yellow arrow heads) by IF and telomeres stained in red by FISH in healthy lung tissue (C) and in tumors (E). Auto-fluorescent red blood cells lacking nuclei are observed in the images. Tumor samples do not show any SFTPC positive cells. Error bars represent standard error. *t*-test was used for statistical analysis. The number of mice are indicated in each case. *, p<0.05. **, p<0,01.

**Fig 6:**
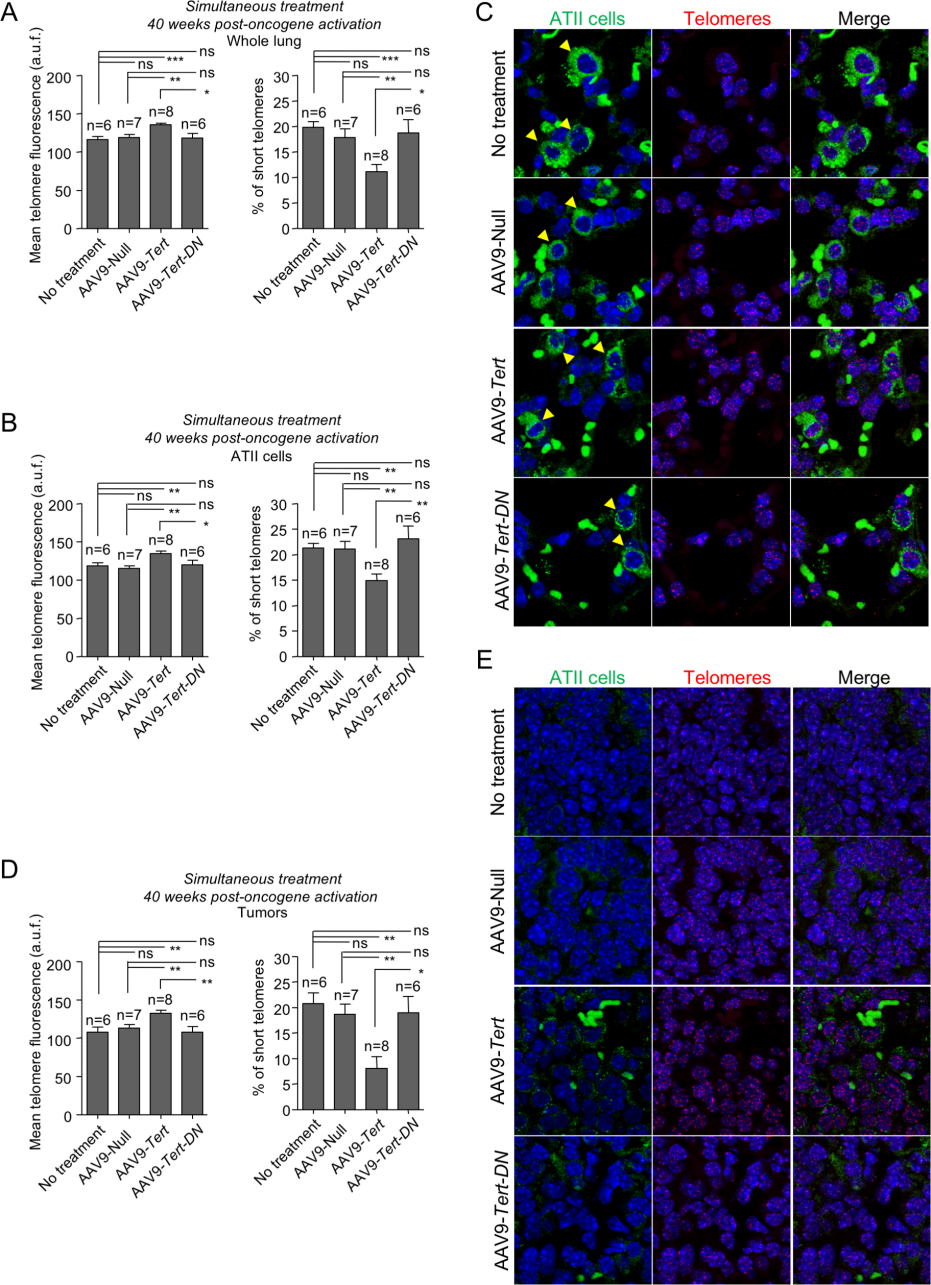
Telomerase gene therapy results in longer telomeres in lung cells and tumors in the simultaneous treatment group. **A-E** Mean telomere fluorescence and percentage of short telomeres in whole lung (A), in ATII cells (B) and in tumors (D) at 40 weeks post-oncogene activation in simultaneously treated mice. **C,E** Representative images showing ATII cells stained with anti-SFTPC (green cytoplasm, yellow arrow heads) by IF and telomeres stained in red by FISH in healthy lung tissue (C) and in tumors (E). Auto-fluorescent red blood cells lacking nuclei are observed in the images. Tumor samples do not show any SFTPC positive cells. Error bars represent standard error. *t*-test was used for statistical analysis. The number of mice are indicated in each case. *, p<0.05. **, p<0,01.

Telomere fluorescence was also measured in healthy whole-lung tissue, in ATII cells, and in tumors at the experimental end-point (40 weeks post-oncogene activation) in both pre-treated (**Fig 5**) and simultaneously treated groups (**Fig 6**). At the end-point, AAV9-*Tert* treated mice in both groups, “pre-treatment” and “simultaneous”, showed longer telomeres and lower percentage of short telomeres both in the whole lung and in ATII cells compared to untreated, AAV9-Null and to AAV9-*Tert-DN* control groups (**Fig 5A–5C** and **Fig 6A–6C**). When telomere length was determined in tumors, we also observed a significant increase in average telomere length and a decrease in the percentage of short telomeres in AAV9-*Tert* treated samples compared to untreated, AAV9-Null and to AAV9-*Tert-DN* control groups (**Fig 5D and 5E; Fig 6D and 6E)**.

### AAV9-*Tert-DN* treatment induces DNA damage and apoptosis and blocks proliferation in lung tumors

**Fig 7:**
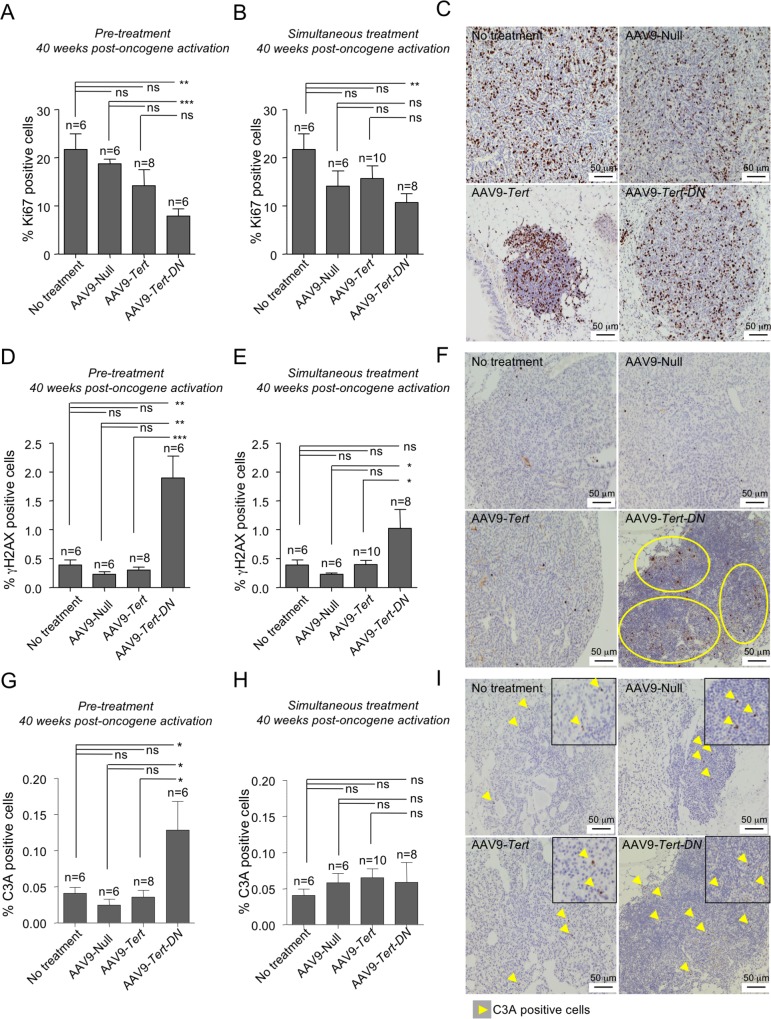
Effect of AAV9 treatments on tumor proliferation DNA damage burden and apoptosis. **A-C** Quantification of the percentage of Ki67 positive cells in pre-treated (A) and simultaneously treated mice (B) at 40 weeks post-oncogene activation; Ki67 representative images correspond to pre-treated mice lung tumors (C). **D-F** Quantification of the percentage of γ-H2AX positive cells in the pre-treated (D) and simultaneously treated (E) mice at 40 weeks post-oncogene activation; γ-H2AX representative pictures correspond to pre-treated mice lung tumors (F). **G-I** Quantification of the percentage of active caspase-3 (C3A) positive cells in pre-treated (G) and simultaneously treated mice (H) at 40 weeks post-oncogene activation; C3A positive cells (yellow arrow heads) in representative images corresponding to pre-treated lung tumors (I). Error bars represent standard error. *t*-test was used for statistical analysis. The number of mice is indicated in each case. *, p<0.05. **, p<0,01. ***, p<0,001.

To understand at the molecular level the impact of different AAV9 treatments on lung tumorigenesis, we next determined DNA damage (γH2AX-positive cells), apoptosis (C3A-positive cells), and proliferation (Ki67-positive cells) in tumors at 40 weeks post-oncogene activation both in the “pre-treatment” and “simultaneous treatment” groups by using immunohistochemistry. Interestingly, in both experimental settings, tumors appearing in the AAV9-*Tert-DN* treated mice showed significantly less Ki67-positive cells compared to AAV9-*Tert* treated and to control groups (no viral treatment and mice treated with AAV9-Null) (**Fig 7A**), which is in agreement with significantly less tumors in this group (**Fig 1D and 1E**). Importantly, no significant differences in Ki67-positive cells were observed between AAV9-*Tert* treated and control mice (no viral treatment and mice treated with AAV9-Null) neither in the “pre-treatment” nor in the “simultaneous” groups (**Fig 7A–7C**), which is in agreement with similar tumor burdens in these groups (**Fig 1G and 1H**).

Next, we determined the impact of different AAV9 treatments on DNA damage induction by quantifying the percentage of γH2AX positive cells in the tumors at 40 weeks after oncogene-induction both in the “pre-treatment” and “simultaneous treatment” groups. We found that lung tumors appearing in the mice with AAV9-*Tert-DN* presented increased number of cells with DNA damage compared to AAV9-*Tert* treated and control mice (no viral treatment and treated with AAV9-Null). Again, the tumors appearing in mice treated with AAV9-*Tert* showed a similarly low abundance of cells positive for γH2AX to the untreated and AAV9-Null treated cohorts (**Fig 7D–7F**).

In accordance with higher DNA damage in tumors from mice pre-treated with AAV9-*Tert-DN* vectors, we observed increased numbers of apoptotic cells in the AAV9-*Tert-DN* treated mice compared to AAV9-*Tert* treated mice and control mice (untreated and AAV9-Null treated cohorts) (**Fig 7G and 7I**). Again, in the “pre-treatment” group we found no significant differences in the number of apoptotic cells between the AAV9-*Tert* and control mice (**Fig 7G and 7I**). However, no differences in the number of apoptotic cells were observed in the “simultaneous treatment” among the different mouse cohorts (**Fig 7H and 7I**), in agreement with similar tumor burden in all cohorts within the simultaneous treatment group (**Fig 1G–1H**)

Altogether, these results indicate that tumor burden in the different cohorts correlates with proliferation, DNA damage and apoptosis, namely tumors appearing in the AAV9-*Tert-DN* “pre-treatment” group have less proliferation and more DNA damage and apoptosis. Importantly, telomerase overexpression in the AAV9-*Tert* treated mice did not influence any of these parameters either in the “pre-treatment” or in the “simultaneous treatment” groups, in agreement with the fact that AAV9-*Tert* treatment did not increase lung tumorigenesis.

### AAV9-*Tert-DN* treatment induces telomeric DNA damage in KRas-induced lung tumors

**Fig 8:**
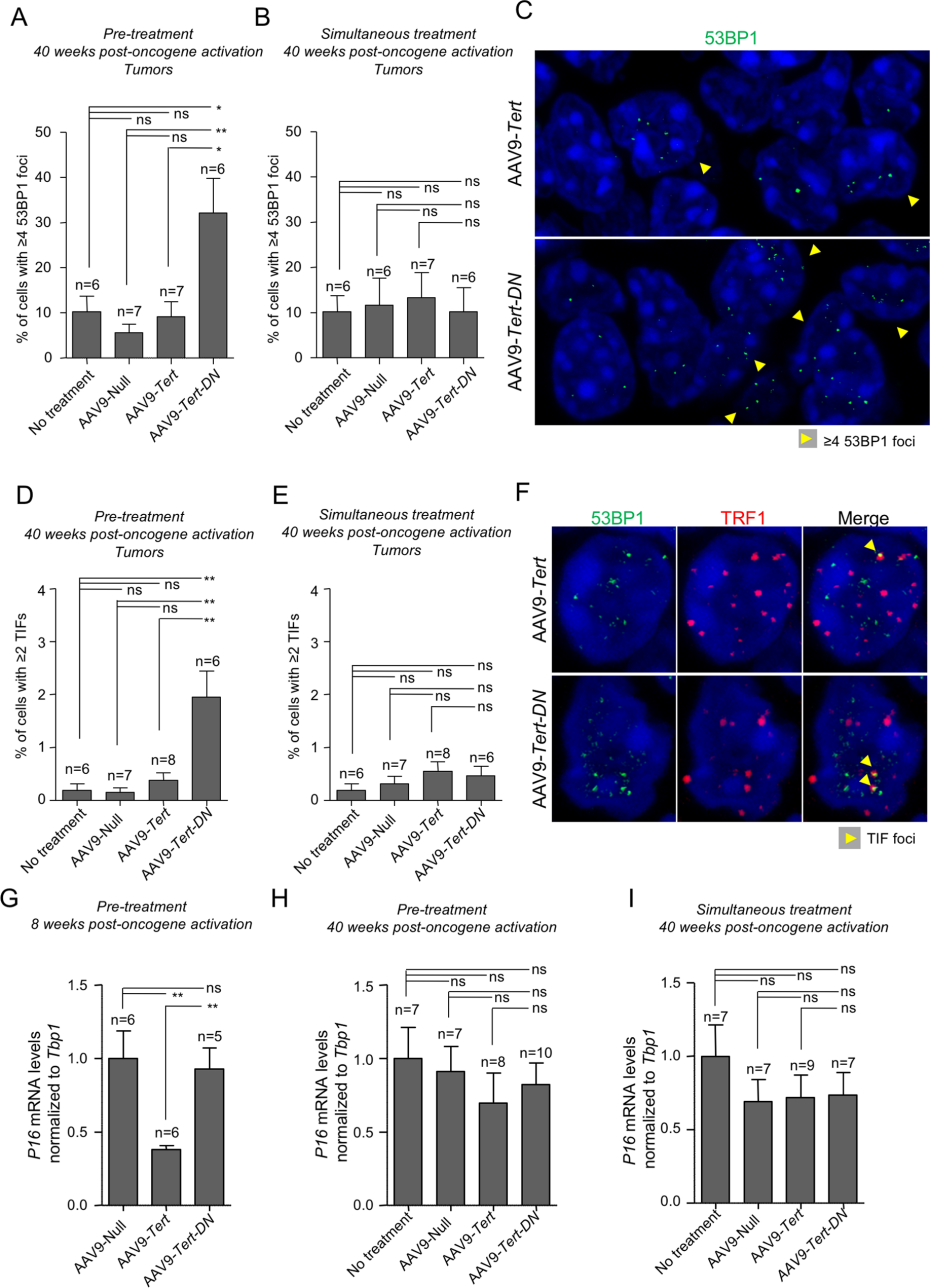
Increased DNA damage after AAV9-*Tert-DN* gene therapy correlate with more telomeric DNA damage. **A-B** Quantification of percentage of cells with more than 3 53BP1 foci by IF, 40 weeks post-oncogene activation in the “pre-treatment” group (A) and in the “simultaneous” group (B). **C** Representative images of 53BP1 staining. Cells with more than 3 53BP1 foci corresponding to pre-treated mice are marked with a yellow arrow head. **D-E** Quantification of percentage of cells with more than 1 telomeric induced foci (TIF) by IF, 40 weeks post-oncogene activation in the “pre-treatment” group (D) and in the “simultaneous” group (E). **F** Representative images of TIF (yellow arrow heads) positive cells corresponding to pre-treated mice. DNA damage and telomeric foci were labeled with antibody against 53BP1 (green) and antibody against TRF1 (red). **G-I** *p16* expression levels measured by Q-PCR in healthy lungs of pre-treated mice at 8 weeks post-oncogene activation (G) and at 40 weeks post-oncogene activation in the pre-treated (H) and in simultaneously treated (I) mice. Error bars represent standard error. *t*-test was used for statistical analysis. The number of mice is indicated in each case. *, p<0.05. **, p<0,01.

In order to study the effects of the different gene therapy vectors on DNA damage specifically located at telomeres, we performed a double immunofluorescence using antibodies against 53BP1 to mark DNA damage foci and TRF1 to mark telomeres in lung tumor sections. To this end, we quantified the number of cells presenting ≥4 53BP1 foci within the tumor in the “pre-treatment” and “simultaneous treatment” groups (**Fig 8A–8C**). In the “pre-treatment” group, tumors appearing in the AAV9-*Tert-DN* treated mice showed a 5-fold increase in 53BP1-positive cells compared to tumors appearing in untreated mice or mice treated with AAV9-Null and AAV9-*Tert* vectors (**Fig 8A**). In the “simultaneous” group, however, no differences in the percentage of damaged cells among the four mouse cohorts were detected (**Fig 8B**). In addition, the percentage of damaged cells presenting ≥ 2 telomere induced foci (TIF) was 5-fold higher in tumors from the AAV9-*Tert-DN* treated group compared to untreated, AAV9-Null and AAV9-*Tert* treated cohorts in the “pre-treatment” group (**Fig 8D**). In the “simultaneous treated group”, however, no differences in the percentage of cells presenting ≥ 2 TIFs among the four mouse cohorts were detected (**Fig 8E**). These results suggest that telomerase inhibition by using AAV9-*Tert-DN* gene therapy vectors previous to oncogene activation results in increased telomere damage associated to oncogenic K-Ras tumorigenesis. This is in agreement with the reduced tumor burden observed in mice in which telomerase activity was inhibited by AAV9-*Tert-DN* before oncogene activation (**Fig 1C–1E and Fig 2C**). The observation that AAV9-*Tert* treatment leads to similar numbers of tumor cells with telomere damage (TIFs) than the AAV9-Null controls, underlies the non-oncogenic effects of telomerase gene therapy.

### AAV9-*Tert* treatment reduces mRNA levels of the p16 senescence marker

Previous studies showed that K-ras mediated lung carcinogenesis induces senescence in the pre-neoplastic lesions (adenomas) and that this senescence is overcome in the more aggressive lesions [52–55]. Interestingly, short/dysfunctional telomeres have been proposed to be at the origin of this oncogene-induced senescence [56, 57]. We set to assess whether treatment with the AAV9-*Tert* and AAV9-*Tert-DN* vectors had any effects on the levels of the senescence marker p16 compared to untreated mice and to mice treated with AAV9-Null. To this end, we determined p16 mRNA levels at 8 and at 40 weeks post-oncogene activation by using q-PCR (**Fig 8G–8I**) [58]. Interestingly, in the “pre-treatment” group, AAV9-*Tert* treated lungs showed significantly lower p16 expression compared to AAV9-*Tert-DN* treated mice and to both untreated and AAV9-Null treated mice at 8 weeks post-oncogene activation (**Fig 8G**), suggesting that telomerase expression previous to oncogene activation inhibits senescence induction at early times during K-Ras induced lung carcinogenesis. However, at 40 weeks post-oncogene activation these differences were lost and we observed similar p16 mRNA expression levels in the different mouse cohorts both in the “pre-treatment” and in the “simultaneous” group (**Fig 8H and 8I**), most likely owing to the more advanced tumor stage. These results indicate that pre-treatment with AAV9-*Tert* leads to reduced senescence associated to oncogene-activation, however, this does not seem to be important for overall tumor progression and aggressiveness.

## Discussion

The majority of adult cell types in both humans and mice do not express telomerase and this results in progressive shortening of telomeres and increased telomere-associated DNA damage with aging [4–6, 13, 21, 58–64], a phenomenon that is proposed to be among the primary causes of organismal aging [11]. Mice are born with longer telomeres than humans but the rate of telomere shortening in blood cells from mice is 100-fold higher than in humans and telomeres do shorten very significantly during the mouse lifespan [14, 21]. Thus, although mice have on average longer telomeres than humans, they also suffer telomere shortening with aging, and indeed this shortening is relevant for aging [13]. In contrast to telomere shortening in healthy cells, the majority of cancer cells aberrantly reactivate telomerase to achieve unlimited proliferative potential, one of the hallmarks of cancer [65]. Indeed, many different human cancer types show mutations in either the promotor region or coding regions of the telomerase catalytic subunit *TERT* gene [23–31, 66]. Also, a wide variety of mouse cancers activate telomerase [32–34]. This frequent re-activation of telomerase in cancer cells, has led to the idea that the postnatal suppression of telomerase activity in the adult organism may have a role as a tumor suppression mechanism. However, there is mounting evidence that short telomeres can also lead to increased cancer, especially owing to accumulation of chromosome aberrations if DNA damage checkpoints are lost, such as p53 loss [61, 67, 68]. Indeed, cancer incidence increases with aging and it is also more elevated in the so-called telomere syndromes [69]. Interestingly, constitutive telomerase expression in telomerase transgenic mouse models shows that telomerase does not act as an oncogene and only leads to slightly higher incidence of some spontaneous cancer at old ages [36, 37]. More recently, we have demonstrated that telomerase activation using non-integrative gene therapy vectors (adeno associated vectors) does not lead to increased cancer both in the context of normal physiological aging [6] and in the context of mouse models of disease including heart infarct [44], aplastic anemia associated to short telomeres [45], and pulmonary fibrosis owing to short telomeres and low doses of a damaging agent to the lungs [46]. Furthermore, we recently demonstrated that chimeric mice with a high percentage of cells having much longer telomeres than those that are normal for the species are in fact cancer protected in agreement with the fact that these mice accumulate less DNA damage with aging, thus suggesting that maintaining long telomeres during aging protect from cancer [70]. However, whether telomerase activation can lead to more cancer in the context of tumor prone contexts remained to be formally addressed.

Here, we demonstrate that telomerase gene therapy does not affect (either increasing or decreasing) tumorigenesis in a well-established mouse model of lung carcinogenesis induced by oncogenic K-ras even in a p53-defficient background [47]. We demonstrate this by using two independent strategies, one in which we over-express *Tert* before the induction of the oncogene and another in which we activate *Tert* at the same time that we induce the oncogenic *K-Ras* allele. This result suggests that endogenous telomerase activation associated to oncogene-induced tumorigenesis is sufficient to allow carcinogenesis and that extra telomerase activation provided by the gene therapy vectors does not affect tumor initiation. Interestingly, when we treated mice with gene therapy vectors carrying a catalytically dead mutant allele of *Tert* that is unable to catalyze the addition of new telomeric repeats, we found a decreased cancer incidence but only when the vector carrying the catalytically dead *Tert* mutant was administered prior to oncogene activation. This is in agreement with previous finding from our group showing that catalytically dead *Tert* acts as a dominant negative blocking endogenous telomerase activity and inhibiting cancer cell growth [51]. Furthermore, we show here that expression *Tert* dominant negative prior to oncogene activation results in lower proliferation and increased DNA damage and apoptosis, thus contributing to block initiation of *K-Ras* carcinogenesis. Although we cannot rule out that AAV9-*Tert-DN* might also have some telomere-independent mechanism to suppress tumor growth, however, the fact that the tumors appearing in mice transduced with AAV9-*Tert-DN* showed a five-fold increase in telomere induced foci (TIFs) clearly indicates that *Tert-DN* expression suppress tumor growth by inducing telomere damage. Of note, this did not occur in the simultaneous treatment group, suggesting that telomerase inhibition once the tumor is already induced has less effect blocking tumorigenesis than when telomerase is inhibited prior to oncogene induction most likely owing to the fact that endogenous telomerase is activated with tumorigenesis.

The tumor suppressive effects observed with AAV9-*Tert-DN* treatment prior to oncogene induction are remarkable since telomerase activity is dispensable for transformation of cells with long telomeres [71]. Indeed, telomerase abrogation in the context of cancer-prone mouse models, including the *K-Ras*^*+/G12D*^ lung tumorigenesis mouse model, only showed anti-tumorigenic activity after several mouse generations in the absence of telomerase when telomeres reached a critically short length [67, 72–74]. However, the fact that AAV9-*Tert-DN* treatment prior to oncogene activation significantly delays tumor onset and progression by increasing telomere-induced DNA damage and apoptosis suggests that telomere length is rate limiting in the early steps of oncogene-induced lung tumorigenesis in mice. These results open new therapeutic opportunities using AAV9-*Tert-DN* gene therapy to prevent tumor induction in cancer prone settings.

Finally, we make here the very intriguing finding that AAV9-*Tert* treatment prior to oncogene induction significantly reduced the levels of the p16 senescence marker. It was been previously proposed that DNA damage associated to oncogene-induced senescence is largely produced by short/dysfunctional telomeres [57]. Our results support this notion as pre-treatment with telomerase is able to significantly decrease senescence in the early steps of lung carcinogenesis. Nevertheless, our results also indicate that senescence does not seem to correlate with tumor burden, as AAV9-*Tert* treatment does not impact on the final number of adenomas and carcinomas.

A limitation for any gene therapy as a treatment for human diseases is the potential immunogenic response elicited either by the viral particles or by the product encoded by the transgene. We use, however, AAV vectors that are weak immunogens [75]. Our AAV9 vectors do not carry any viral gene and no immunogenic response against AAV9 have been reported in mice [76]. In line with this, we did not observe a significant difference in the tumor burden between the untreated and the AAV9-null treated mice, although there was a trend to lower tumor burden in the mice that received the AAV9 vectors in some of the parameters measured. Regarding the potential immune response against the AAV9-encoded transgene product, namely telomerase, we think it is unlikely since the transgene encodes the endogenous mouse telomerase. In agreement with this, we found no significant difference in the tumor burden between the AAV9-*Null* and the AAV9-*Tert* treated mice. Indeed, we have tested the AAV9-*Tert* gene therapy in several previous works and never observed an immunogenic effect of mouse *Tert* in mice [6, 44–46]. However, the potential immunogenic response of human telomerase in humans deserves further clinical research.

The experimental design of this work poses the limitation that the delivery of the Cre recombinase to induce oncogenic K-Ras expression and of telomerase were performed using different viral vectors, adeno virus and adeno associated virus, respectively. Hence, there was no mechanism to select for cells that were infected with both virus. Furthermore, owing to the packaging limit of AAV vectors to 5 kilobases [77], it was not possible to carry both the Cre and the *Tert* genes in the same AAV9-vector in the “simultaneous strategy”. Nevertheless, we observed that the K-Ras expressing tumors also expressed the AAV9-transduced *Tert/Tert-DN* genes, indicating that the majority of the tumors originated from cells transduced with the AAV9 vectors. Furthermore, we show that one week after co-infection with adeno-Cre and with AAV9-GFP the β-gal positive cells also expressed GFP, demonstrating that lung cells are susceptible to be co-infected with adeno and adeno-associated virus. However, we cannot rule out the possibility that some tumors arise from cells only infected by the adeno-Cre and not by the AAV9 vectors.

One could argue than in a long-lived specie like humans, the exogenous expression of *Tert* in a wild-type scenario may take several years/decades to facilitate cancer development, the time needed to acquire additional oncogenic mutations that eventually lead to malignant transformation and tumor development. To model the impact of AAV9-*Tert* in cancer in short lived mice, we forced oncogenic Ras expression to induce tumorigenesis both in wild-type and p53-null genetic backgrounds, thereby avoiding this lagging time. In addition, to avoid that long-term expression of telomerase could facilitate cancer development after several decades, we use non-integrative AAV9 vectors, which allow only for a transitory expression of telomerase due to the fact that as cells divide the virus load is progressively diluted until eventually cells lose the expression of the transgene (*Tert*).

In summary, the results shown here demonstrate that telomerase activation by adeno associated vectors does not increase lung carcinogenesis even in the context of an activated *K-Ras* oncogene, highlighting the safety of therapeutic strategies based on telomerase activation using AAV9 vectors.

## Methods

### Mice

*K-Ras*^*+/G12V*^ mice were generated as previously described [47]. *K-Ras*^*+/G12V*^ mice were crossed with *p53*^-*/-*^ mice (Jackson Labs, http://jaxmice.jax.org/strain/002101.html) to generate the compound *K-Ras*^*+/G12V*^ *p53*^-*/-*^ mouse. Separated groups of mice were tail-vein injected with 2x10^12^ vg (viral genomes)/mouse of either AAV9-Null, AAV9-*Tert* or AAV9-*Tert-DN*, a catalytically inactive form of mouse TERT. All mice were maintained at the Spanish National Cancer Centre under specific pathogen-free conditions in accordance with the recommendations of the Federation of European Laboratory Animal Science Associations (FELASA). All animal experiments were approved by the Ethical Committee (CEIyBA) and performed in accordance with the guidelines stated in the International Guiding Principles for Biomedical Research Involving Animals, developed by the Council for International Organizations of Medical Sciences (CIOMS).

### Gene therapy vector production

Adeno-associated viral vectors (AAV9) were generated and purified as previously described [78]. The vectors used were (i) AAV9-Null (ii) AAV9-*Tert* that express murine catalytic subunit of telomerase (iii) AAV9-*Tert-DN* that express murine catalytically inactive telomerase (iv) AAV9-*GFP* [6]. AAV9 particles were purified using 2 cesium chloride gradients, dialyzed against phosphate-buffered saline (PBS) and filtered. Viral genome particle titers were determined by a quantitative real-time polymerase chain reaction (PCR) method.

### Adenovirus intratracheal infection

Twelve-week-old mice were treated once with intratracheal adeno-Cre vectors (Gene Vector Core, University of Iowa, 1x10^10^ pfu/ml) instillation with 1x10^8^ pfu/mouse of virus after anesthesia by intraperitoneal injection of ketamine-medetomidine (Domitor, 1mg/ml; Orion Corporation). To wake up the mice after the instillation, they were injected with 0.05 mg of atipamezole (Antisedan, 5mg/ml; Orion Corporation).

### *In vivo* imaging by computed tomography (CT)

Eight weeks after adeno-Cre inoculation, an *in vivo* follow-up of tumor growth was achieved by four computed tomographies (CT) every 8 weeks. CT analyses were performed as previously described [79]. Briefly, Micro‐CT imaging was performed on a high resolution scanner (Locus, General Electric HealthCare, London,Ontario,Canada). The scanning protocol operates at 80 kVp and 50 mA, 400 projections and collected in one full rotation of the gantry in approximately 10, minutes. The reconstruction was done with a modified Feldkamp cone‐beam algorithm. Micro‐CTimages were analyzed using MicroView Analysis + (v2.2, General Electric Healthcare, London, Ontario, Canada).

### Immunohistochemistry analyses in tissue sections

Lungs were fixed in 10% buffered formalin, embedded in paraffin wax and sectioned at 5 mm. For pathological examination sections were stained with hematoxylin and eosin, according to standard procedures. Antibodies used for immunohistochemistry in lung tumor sections included those raised against: γH2AX Ser 139 (Millipore), Ki67 (Master diagnostica), C3A (Cell Signaling Technology), β-GAL (3A9A; CNIO Monoclonal Antibodies Core Unit, AM(3A9A)) and GFP (Cell Signaling). For β-GAL and GFP double staining, the immunohistochemical reaction was developed using 3,30-diaminobenzidine tetrahydrochloride (DAB) (Chromomap DAB, Ventana, Roche) and purple chromogen (Discovery Purple Kit, Ventana, Roche), respectively. Nuclei were counterstained with Harrys’s hematoxylin. Pictures were taken using Olympus AX70 microscope.

### Real-time qPCR

Total RNA from cells was extracted with the RNeasy kit (QIAGEN) and reverse transcribed was using the iSCRIPT cDNA synthesis kit (BIO-RAD) according to manufacturer’s protocols. Quantitative real-time PCR was performed with the QuantStudio 6 Flex (Applied Biosystems, Life Technologies) using Go-Taq qPCR master mix (Promega) according to the manufacturer’s protocol. Samples were run in triplicates. Primers used are as follows: TBP1-F 5’-ACCCTTCACCAATGACTCCTATG-3’; TBP1-R 5’-TGACTGCAGCAAATCGCTTGG-3’; TERT-F 5’-GGATTGCCACTGGCTCCG-3’; TERT-R 5’-TGCCTGACCTCCTCTTGTGAC-3’; P16-F 5’-TACCCCGATTCAGGTGAT; P16-R 5’-TTGAGCAGAAGAGCTGCTACGT-3’; CMV-F 5’-CAATTACGGGGTCATTAGTTCATAGC-3’.

### Telomere length analyses on tissue sections

Quantitative telomere fluorescence *in situ* hybridization (Q-FISH) was performed directly on parafinn-embedded lung sections as previously described [21] and analysed by Definiens software. The incidence of short telomeres was calculated as the percentage of telomeres below the 25^th^ percentile of telomere fluorescence in AAV9-null samples.

### Immunofluorescence analyses on tissue sections

For immunofluorescence analyses, tissue sections were fixed in 10% buffered formalin (Sigma) and embedded in paraffin. After desparaffination and citrate antigen retrieval, sections were permeabilized with 0.5% Triton in PBS and blocked with 1%BSA and 10% Australian FBS (GENYCELL) in PBS. The antibodies were applied overnight in antibody diluents with background reducing agents (Invitrogen). Primary antibodies: polyclonal rabbit anti-SFTPC (Sigma), rat polyclonal anti-TRF1 (homemade), anti-53BP1 (Novus Biologicals). Immunofluorescence images were obtained using a confocal ultraspectral microscope (Leica TCS-SP5). Quantifications were performed with Definiens software. A double immunofluorescence using antibodies against 53BP1 to mark DNA damage foci and TRF1 to mark telomeres was performed in lung tumor sections to assay for telomeric DNA damage specifically located at telomeres.

## Supporting information

S1 Fig Adeno and adeno-associated virus co-infect lung cells. **A.** Representative images of β-Gal (brown) immunohistochemistry staining of lungs one week after double infection with adeno-cre and AAV9-GFP. **B.** Representative images of β-Gal (brown) and GFP (purple) immunohistochemistry double staining of lungs one week after double infection with adeno-cre and AAV9-GFP. Brown arrow marks β-gal positive cell cluster. Green arrow marks a single AAV9-GFP positive cell. Red arrows mark cluster of cells double positive for β-Gal and GFP.
(TIF)

S2 Fig AAV9-*Tert* therapy does not aggravate *K-Ras*-mediated lung tumor development in a p53-deficient background. **A.** Eight weeks old *K-Ras*^*+/G12V*^ *p53*^-*/-*^ mice were transduced with AAV9 (Null or *Tert*) vectors by tail vein injection and four weeks after they were infected with Adeno-cre intratracheally. Mice were sacrificed 5 months post-oncogene activation for pathological analysis. **B-C** Macroscopic quantification of total number of tumors per mouse (B) and tumor burden according to tumor diameter per mouse (C). Error bars represent standard error. *t*-test was used for statistical analysis. The number of mice are indicated in each case.
(TIF)
