# Supplementary figures and images for: AAV9-mediated telomerase activation does not accelerate tumorigenesis in the context of oncogenic K-Ras-induced lung cancer
Source: PLoS Genet. 2018 Aug 16;14(8):e1007562. doi: 10.1371/journal.pgen.1007562 (PMC6095492; doi:10.1371/journal.pgen.1007562)

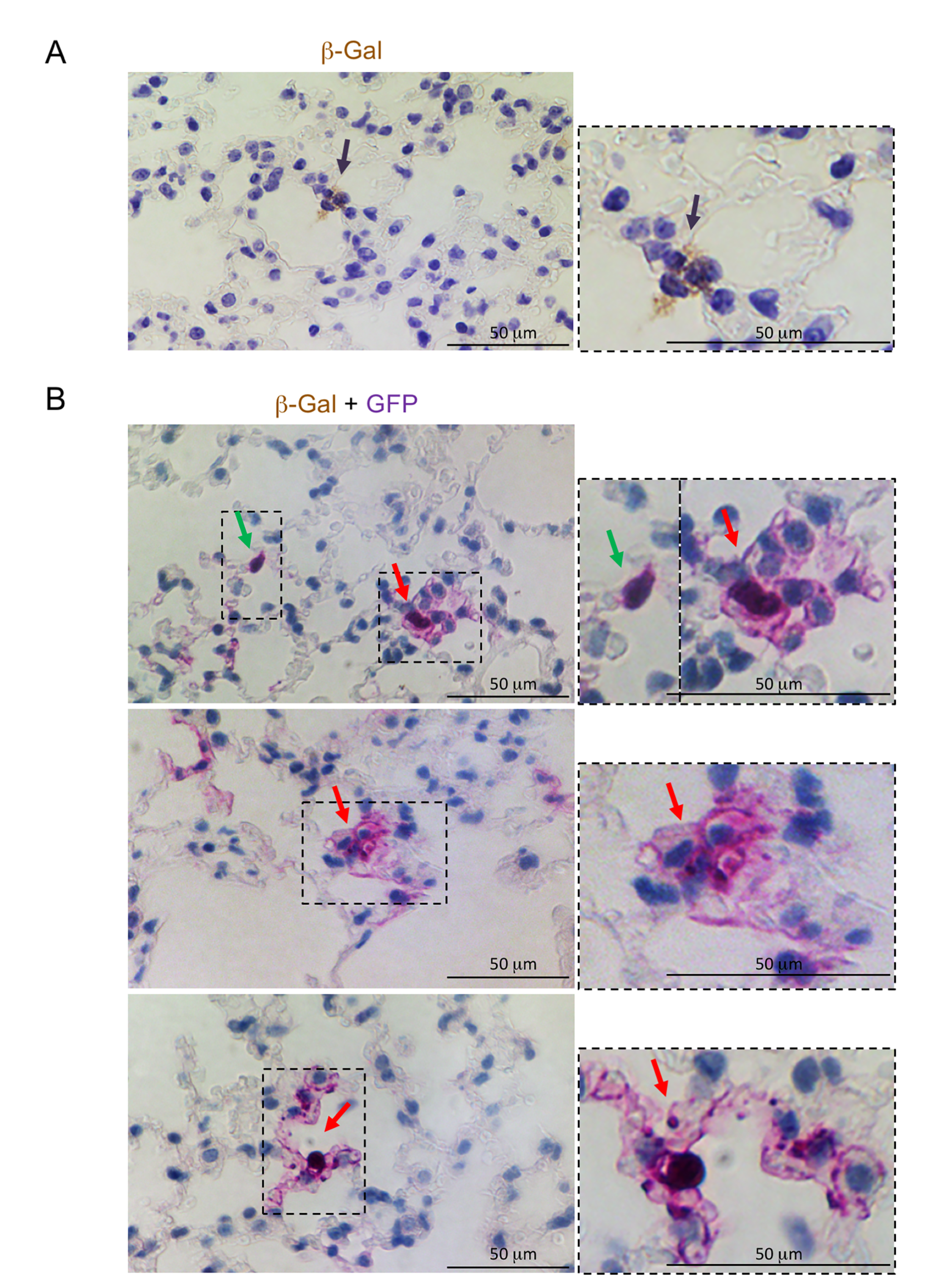

Supplement: S1 Fig — A. Representative images of β-Gal (brown) immunohistochemistry staining of lungs one week after double infection with adeno-cre and AAV9-GFP. B. Representative images of β-Gal (brown) and GFP (purple) immunohistochemistry double staining of lungs one week after double infection with adeno-cre and AAV9-GFP. Brown arrow marks β-gal positive cell cluster. Green arrow marks a single AAV9-GFP positive cell. Red arrows mark cluster of cells double positive for β-Gal and GFP. (TIF) [file pgen.1007562.s001.tif]

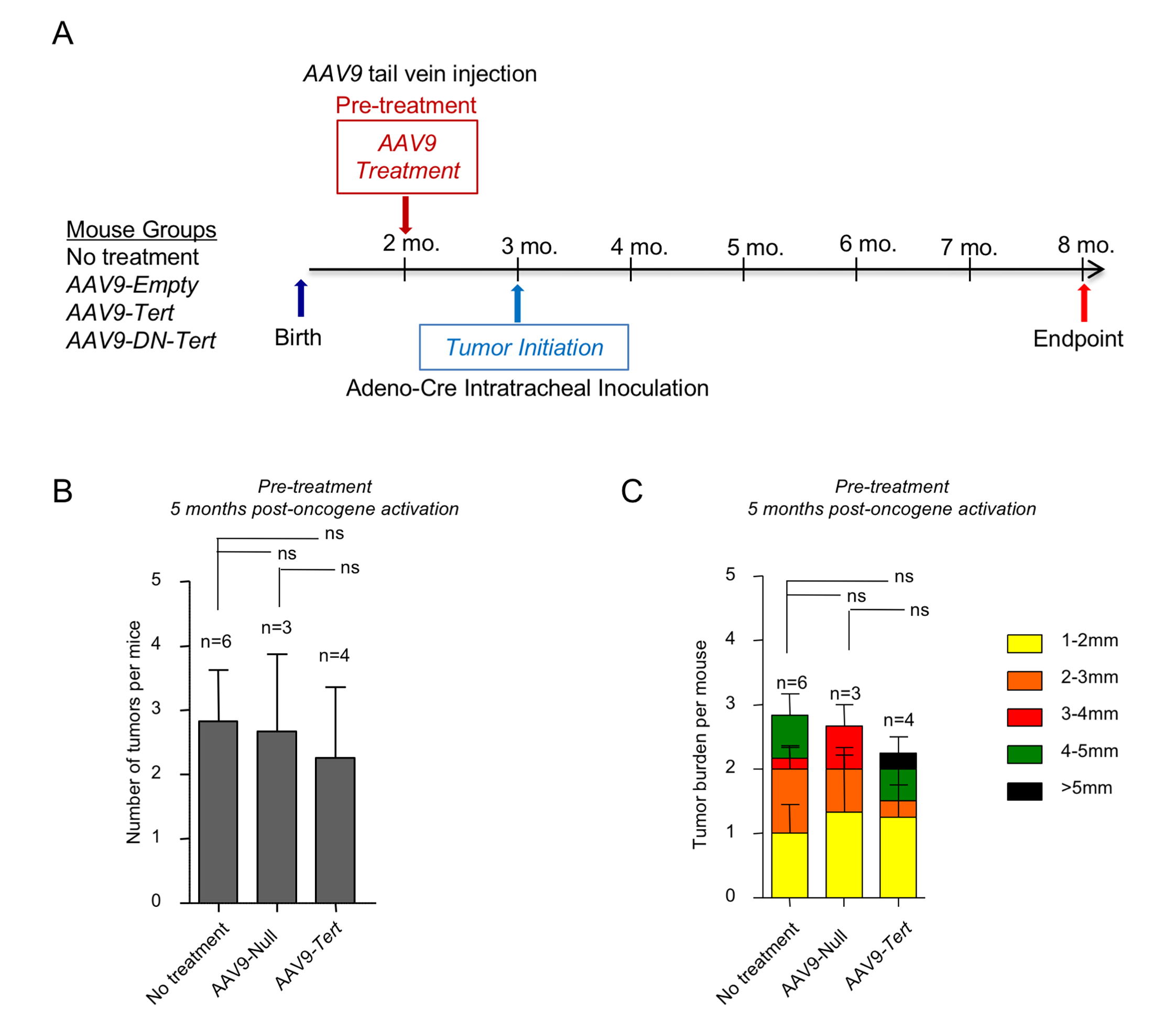

Supplement: S2 Fig — A. Eight weeks old K-Ras+/G12V p53-/- mice were transduced with AAV9 (Null or Tert) vectors by tail vein injection and four weeks after they were infected with Adeno-cre intratracheally. Mice were sacrificed 5 months post-oncogene activation for pathological analysis. B-C Macroscopic quantification of total number of tumors per mouse (B) and tumor burden according to tumor diameter per mouse (C). Error bars represent standard error. t-test was used for statistical analysis. The number of mice are indicated in each case. (TIF) [file pgen.1007562.s002.tif]
